# Effects of Danhong injection on cardiac function and blood lipid in patients with angina pectoris of coronary heart disease: A protocol for randomized, double-blind, placebo-controlled clinical trial

**DOI:** 10.1097/MD.0000000000027479

**Published:** 2021-10-29

**Authors:** Jian Liang, Xiaojiao He, Hongyu Zhou, Peng Liang

**Affiliations:** The People's Hospital of Dazu, Chongqing Dazu, Chongqing Province, China.

**Keywords:** angina pectoris of coronary heart disease, clinical randomized controlled trials, Danhong injection, protocol

## Abstract

**Background:**

Angina pectoris of coronary heart disease is the leading cause of death worldwide. Danhong injection is a supplement for angina pectoris of coronary heart disease. A large number of studies have confirmed its efficacy and safety. However, there is no rigorous clinical study to evaluate the effects of Danhong injection on cardiac function and blood lipid in patients with angina pectoris of coronary heart disease.

**Methods:**

This is a prospective, randomized, double-blind, placebo-controlled trial to study the effects of Danhong injection on cardiac function and lipid profile in patients with angina pectoris of coronary heart disease. Participants will be randomly divided into treatment group and control group. The treatment group will be treated with Danhong injection and the control group will be treated with placebo under basic treatment according to recommended guideline, and followed up for 3 months after 14 consecutive days of treatment. Outcomes include: cardiac function (left ventricular end-diastolic diameter); left ventricular end-systolic diameter; left ventricular ejection fraction, blood lipid levels (total cholesterol; triacylglycerol; low density lipoprotein cholesterol; high density lipoprotein cholesterol), the number of angina attacks per week, total amount of nitroglycerin tablets, and adverse reactions.

**Discussion:**

This study will evaluate the efficacy of Danhong injection in improving cardiac function and blood lipid in patients with angina pectoris of coronary heart disease. The results of this study will provide reference for clinical use of Danhong injection to improve cardiac function and blood lipid in patients with angina pectoris of coronary heart disease.

Trial registration: OSF Registration number: DOI 10.17605/OSF.IO/TPZJ5.

## 1 Introduction

Cardiovascular and cerebrovascular diseases are the main causes of death worldwide.^[1]^ According to WHO statistics, 17.9 million people died of cardiovascular diseases worldwide in 2016, accounting for 31% of the total global deaths, among which about 34% died of coronary heart disease.^[2]^ Coronary heart disease has become 1 of the diseases with the highest mortality rate in human beings and seriously endangers human physical and mental health.^[1]^ Angina pectoris of coronary heart disease is a syndrome caused by acute myocardial ischemia and anoxia,^[3]^ its pathogenesis is complex and generally related to epicardial coronary artery stenosis caused by atherosclerosis, reducing the coronary circulation of myocardial blood supply. And coronary microcirculation, imbalance between myocardial oxygen supply and metabolic oxygen demand are also important influence factors. Its molecular mechanism may involve vascular endothelial function, immune inflammation, thrombosis, lipid metabolism, oxidative damage, and other factors.^[4]^

At present, drug therapy for angina pectoris of coronary heart disease includes nitrates, calcium antagonists, antiplatelet aggregation, drugs that improve myocardial metabolism, drugs that stabilize atherosclerotic plaques and β-blockers, etc.^[5]^ Although some drugs take effect fast and the curative effect is distinct, but in the face of angina pectoris of coronary heart disease, which is a multi-pathway and multi-gene complex disease, the drugs still exist certain limitations.^[6]^ And clinical studies have found that traditional anti-myocardial ischemia therapy does not reduce cardiovascular adverse events, there are still 5% to 15% of patients with refractory angina pectoris attack.^[6,7]^

Traditional Chinese medicine has the characteristics of multi-target and multi-link, especially in the treatment of cardiovascular diseases with a long history, and its efficacy and safety have been widely studied and verified.^[8]^ Traditional Chinese medicine treatment schemes vary with different pathogenesis. Blood stasis syndrome is usually the main pathogenesis of angina pectoris of coronary heart disease, so activating blood circulation and removing blood stasis is a very important therapeutic goal.^[9]^ Danhong injection, consisting of Dan Shen and Hong Hua, is a traditional Chinese medicine injection based on promoting blood circulation and removing blood stasis, which is widely used in the treatment of cardiovascular diseases in China,^[10]^ and strongly recommended in *the Clinical Application Guide of Chinese Patent Medicine in treating Coronary Heart Disease (2020)*.^[11]^ Pharmacological studies have found that Danhong injection has effects of anti-inflammatory, antioxidant, anticoagulant, lipid-lowering, anti-apoptosis, vasodilation, and angiogenesis promotion.^[12]^ Clinical studies have confirmed that Danhong injection can relieve angina symptoms and improve electrocardiogram.^[13]^ Although current studies have confirmed the effectiveness and safety of Danhong injection in the treatment of angina pectoris of coronary heart disease,^[14]^ there is no study on the effect of Danhong injection on the cardiac function and blood lipid of the limbs affected by angina pectoris of coronary heart disease. The changes of cardiac function and blood lipid in patients with angina pectoris of coronary heart disease are of great significance to evaluate the prognosis of patients, which can also reflect the long-term efficacy of drugs. This study will explore the effects of Danhong injection on cardiac function and blood lipid in patients with angina pectoris of coronary heart disease in a prospective, randomized, double-blind, placebo-controlled trial.

## 2 Materials and methods

### 2.1 Study design

**Figure 1:**
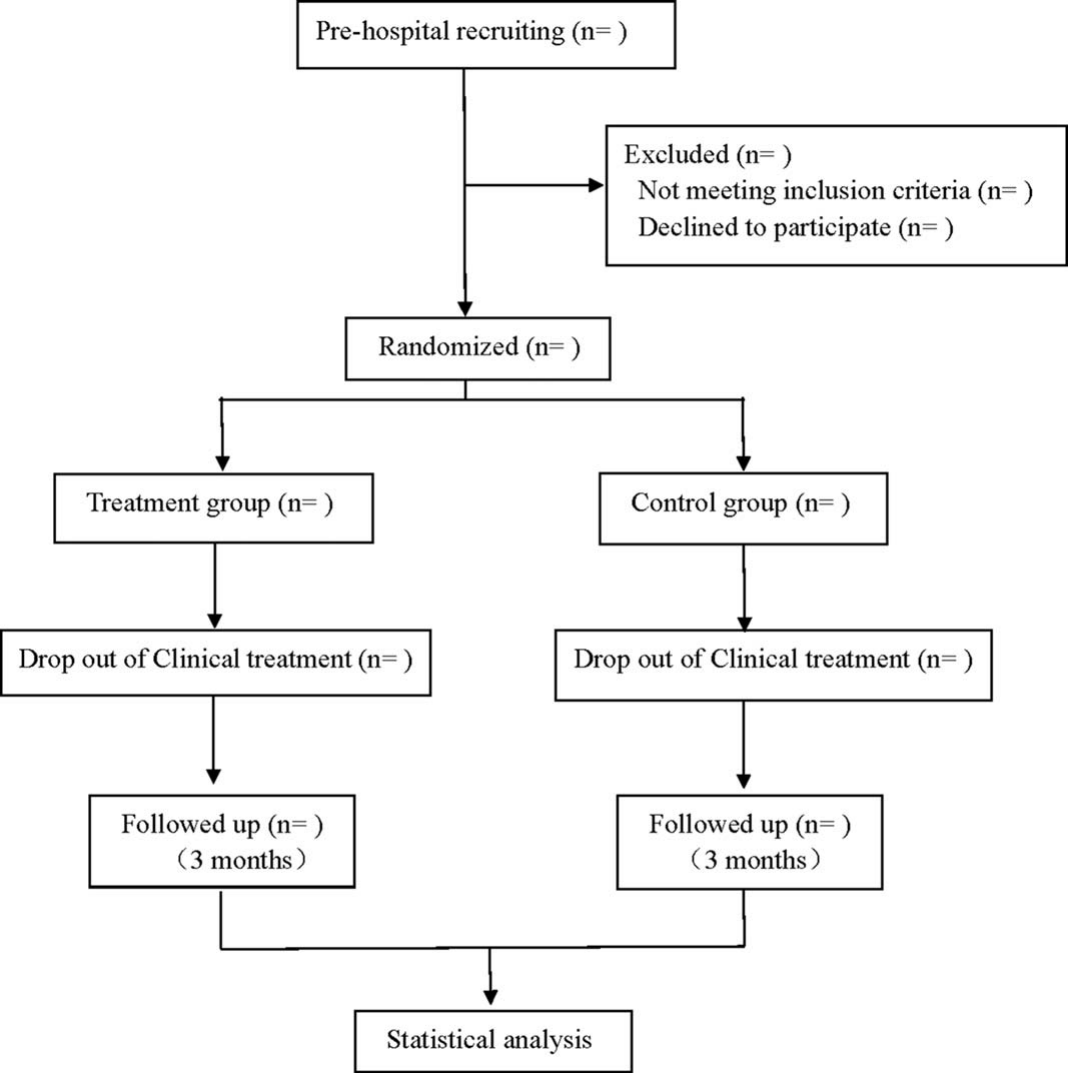
Flow diagram of this clinical trial.

This is a prospective, randomized, double-blind, placebo-controlled study of the effects of Danhong injection on cardiac function and lipid profile in patients with angina pectoris of coronary heart disease. Participants will be randomly divided into treatment group and control group, the treatment group will receive Danhong injection treatment, and the control group will receive placebo treatment. The patients will be followed up for 3 months after 14 days of continuous treatment. Flow diagram is shown in Figure 1, study schedule is shown in Table 1. This protocol followed the latest Consolidated Standards of Reporting Trials 2017 and Standard Protocol Items: Recommendations for Interventional Trials 2013 statement.

**Table 1:** Study schedule.

	Screening period	Treatment period		Follow-up		
--Project Stage	Baseline	1-wk	2-wk	1-mo	2-mo	3-mo
Record fill	√					
Fulfill inclusion criteria and exclusion criteria	√					
Sign informed consent	√					
Random allocation	√					
Treatment	√	√	√			
Effectiveness observation						
Cardiac function	√		√			√
Blood lipid levels	√		√			√
The number of angina attacks per week	√	√	√	√	√	√
Total amount of nitroglycerin tablets	√		√			√
Safety evaluation						
Blood test and urinalysis	√		√			√
Liver and kidney function	√		√			√
Record of adverse event		√	√	√	√	√

### 2.2 Ethics and registration

This Research protocol will be conducted in accordance with Declaration of Helsinki and Ethical Guidelines for Clinical Research. This study has been approved by our Clinical Research Ethics Committee and has registered in open science framework (registration number: DOI 10.17605/OSF.IO/TPZJ5). Before randomization, all patients were required to sign an informed consent form, giving them the option of continuing the trial at any time.

### 2.3 Patients

#### 2.3.1 Diagnostic basis

The diagnostic criteria for angina pectoris of coronary heart disease refers to *Nomenclature and criteria for diagnosis of ischemic heart disease*.^[15]^ The diagnostic criteria of TCM diseases and syndromes refers to *Diagnostic and therapeutic criteria of traditional Chinese medicine diseases*.^[16]^

#### 2.3.2 Inclusion criteria

- Patients who meet the diagnostic basis of angina pectoris of coronary heart disease, and meet the requirements of coronary angiography or coronary angiography that there is at least 1 coronary artery stenosis with catheter stenosis ≥50%, and whose myocardial ischemia and ECG ST-T changes are confirmed by cardiac MRI or radionuclide myocardial perfusion imaging.
- Patients whose age ≥18 years, and ≤70 years;
- Patients who agree to participate in this study and signed informed consent.

#### 2.3.3 Exclusion criteria

- Patients who have severe mental illness, severe cardiopulmonary insufficiency, severe arrhythmia, or other serious illness (such as tumor);
- Patients whose ALT, AST or Cr reaches 1.5 times of normal upper limit^[17]^;
- Patients who have other diseases that may increase the risk of bleeding, such as a history of bleeding in vital organs (such as cerebral hemorrhage and upper gastrointestinal bleeding), reduced platelet count, abnormal coagulation function, and recent active bleeding within the last 6 months;
- Women who are pregnant, preparing to pregnant or breastfeeding;
- Patients who are allergic to the drugs used in the study;
- Patients who have participated within the last 1 month or are currently participating in other clinical trials.

#### 2.3.4 Shedding criteria and management

- Patients who appear severe adverse events or other complications and special physiological changes. The trial should be stopped and corresponding treatment should be given according to the judgment of the investigator;
- If the subject's condition does not improve or even deteriorate to a certain extent within a certain period of time, although the required follow-up had not been completed, the subject should be withdrawn from the study and received other effective treatments in order to protect the subject;
- Patients who have poor compliance, change medications or add non-prescribed medications, which may affect the result of the study;
- Patients who are unwilling or impossible to continue the clinical trial for any reason, and suspend the test by requesting the investigator to withdraw from the test;
- Patients who are misdiagnosed, or accidentally included without meeting the inclusion and exclusion criteria.

For patients who dropped out of the trial or lost to follow-up, researchers should take active measures to complete the last test as far as possible, so as to analyze its efficacy and safety, and take corresponding treatment measures. All shedding cases should be recorded on the case report form (CRF), and filled in the cause.

### 2.4 Sample size

The sample size was estimated based on the mean and standard deviation of the left ventricular ejection fraction (LVEF) scores of patients 14 days after treatment. Based on the results of the preliminary experiment, the LVEF score was 53.13 ± 6.18 in the treatment group and 49.78 ± 5.78 in the control group. α = 0.025, one-tailed test, β = 0.20. The PASS15.0 software calculated that 49 participants were required for each group, with an estimated drop-out rate of 10%, 55 patients would be enrolled in each group.

### 2.5 Randomization and blinding

Patients meeting the criteria would be randomly assigned to treatment group (Danhong injection group) or control group (placebo group) in a 1:1 ratio through a randomization tool based on a central network. Random sequences were generated using SAS 9.3 software (SAS Institute, Cary, NC) by independent statisticians that were not involved in trial implementation or statistical analysis. The research assistant entered patient information on a tablet computer and was assigned a random number to complete the random assignment. The outcome of the assignment was unknown to the patients and principal investigator throughout the study. Because Danhong injection was different from 0.9% normal saline in color, we used opaque brown bag to seal the infusion bottle and used brown infusion set for infusion. A professional nurse prepared the medication in a dedicated infusion room and sealed the infusion bottle in a brown bag with only the patient's number on it, another nurse was responsible for completing the infusion according to the number. These programs would be implemented by 2 professional nurses, and they would be asked to sign a confidentiality agreement before the start of the study without being in contact with each other.

### 2.6 Intervention measures

According to *2014 American College of Cardiology/American Heart Association Guidelines for the Diagnosis and Treatment of Non-ST-Segment Acute Coronary Syndrome*, all patients would receive dual antiplatelet therapy (aspirin 100 mg/d +clopidogrel 75 mg/d) and anticoagulant therapy (unfractionated heparin). And they would also receive statins, angiotensin converting enzyme inhibitors, beta-blockers, and nitrates according to the guidelines.^[18]^ All of these basic treatments will be detailed on the patient's medical record and CRF.

The treatment group was treated with Danhong Injection (Danhong Pharmaceutical Co., Ltd., Shandong, China), 40 ml/d combined with 0.9% normal saline, 250 ml/d intravenously, and the control group was treated with placebo (0.9% normal saline), 40 ml/d, combined with 0.9% normal saline, 250 ml/d, intravenously. All patients received standard treatment for 14 days.

When angina attacks, patients were instructed to take nitroglycerin tablets (Yimin Pharmaceutical Co., Ltd., Beijing, China), 0.5 mg/time, repeated every 5 minutes until angina relieved. Timing and dosage would be recorded in detail on the Case Report Form.

### 2.7 Outcomes

- Cardiac function: Color Doppler ultrasonography (GE-VIVID7, US) was used before and after treatment and during follow-up time to detect patient's left ventricular end-diastolic diameter, left ventricular end-systolic diameter, and LVEF.
- Blood lipid levels: Fasting vein blood was collected in the morning before and after treatment and during follow-up time, and automatic biochemical analyzer (HITEC-7100, Japan) was used to detect blood lipid indexes of the patients, including total cholesterol; triacylglycerol; low density lipoprotein cholesterol; high-density lipoprotein cholesterol.
- The number of weekly attacks of angina pectoris; total amount of nitroglycerin tablets taken during the study period.

### 2.8 Safety evaluation

Blood routine tests, urine routine tests, liver function(ALT, AST, DBIL, IBIL, ALP, γ-GT), renal function (BUN, Scr) and ECG were performed at baseline and after treatment to evaluate the safety of treatment. Patients would be asked to report any abnormal reactions to the researchers during the study. Details of all adverse events would be recorded in CRF, including the time, extent and duration of occurrence, suspected reasons, effective measures, and outcomes. After treatment, the incidence of adverse reactions in the 2 groups was analyzed.

### 2.9 Data management and quality control

Any modifications or changes to the protocol will be re-approved through a formal process by the Hospital Ethics Committee. Independent clinical research assistants will periodically review research progress. Study data will be collected and recorded in CRF by trained investigators. To ensure reliability of data, personal information about potential and registered participants will be collected, shared and kept in a separate repository to protect confidentiality before, during, and after the trial. Access to the database will be restricted to the researchers in the research team. Participants’ information will not be open or shared without their written permission.

### 2.10 Statistical analysis

Efficacy evaluation will be determined by full analysis set and per-protocol set, and safety evaluation will be based on safety set. Statistical evaluation of full analysis set will follow intent-to-treat principles. Last observation carried forward method was used to estimate missing values of major variables. The collected data were statistically analyzed by SPSS 22.0 software. Chi-square test was used for enumeration data; mean value ± standard deviation ($\bar{\text{x}}$ ± S) was used for measurement data, independent sample *t* test was used for normal distribution, and Mann-Whitney *U* test was used for skewed distribution. When *P* < .05, the difference was considered statistically significant.

## 3 Discussion

There are many risk factors of angina pectoris of coronary heart disease. Hypertension, diabetes, age, poor dietary habits, obesity and other diseases are all related to this disease, and fatigue, heavy mental burden, and emotional excitement are the main causes of its occurrence.^[19]^ Angina pectoris of coronary heart disease has a great impact on patients’ health and quality of life, so it needs timely treatment.

In patients with coronary heart disease, various pathogenic factors could cause left ventricular thickened, increase diastolic and systolic blood pressure, and increase the ratio of ventricular volume to pressure, leading to a compensatory increase in ventricular septum, left ventricular end-diastolic diameter, left ventricular end-systolic diameter, and left ventricular posterior wall thickness.^[20]^ Patients with angina pectoris of coronary heart disease are often accompanied by obvious dyslipidemia, which is also 1 of the most common and dangerous factors that cause angina pectoris of coronary heart disease.^[21]^ Danhong injection is a certified Chinese patent medicine extracted from Dan Shen and Hong Hua. The experimental results showed that Danhong injection could inhibit the expression of transforming growth factor-β, matrix, metalloproteinases-2, and MMP9, as well as the accumulation of cardiac collagen to alleviate myocardial fibrosis and improve cardiac function by preventing poor myocardial remodeling after coronary artery ischemia.^[22]^

Danhong injection can also reduce the level of blood lipids, inhibit the formation of lipid peroxidation products, regulate lipoprotein metabolism disorder, increase the activity of antioxidant enzymes and inhibit the development of hyperlipidemia.^[23]^ However, these conclusions are based on animal experimental studies, and there is no rigorous clinical study to explore the effects of Danhong injection on the cardiac function of patients with angina pectoris of coronary heart disease. Therefore, this study intends to explore the effects of Danhong injection on cardiac function and blood lipid in patients with angina pectoris of coronary heart disease through a standard randomized controlled trial.

There are also some shortcomings in this study: although our follow-up time is 3 months, which is much longer than that of other clinical studies of Danhong injection, it may still be unable to observe the impact of Danhong injection on major cardiovascular adverse events, and whether Danhong injection has a positive effect on the survival cycle of patients with angina pectoris of coronary heart disease; since the efficacy of Danhong injection was observed on the basis of standard treatment in this study, we are still unable to determine whether Danhong injection can be used as a complete alternative treatment for angina pectoris of coronary heart disease.

## Author contributions

**Conceptualization:** Jian Liang and Hongyu Zhou

**Data curation:** Jian Liang and Xiaojiao He

**Formal analysis:** Xiaojiao He and Hongyu Zhou

**Funding acquisition:** Peng Liang

**Software:** Xiaojiao He and Peng Liang

**Supervision:** Hongyu Zhou and Peng Liang

**Writing – original draft:** Jian Liang and Hongyu Zhou

**Writing – review & editing:** Jian Liang and Xiaojiao He
